# Production, Characterization, and Application of *Bacillus licheniformis* W16 Biosurfactant in Enhancing Oil Recovery

**DOI:** 10.3389/fmicb.2016.01853

**Published:** 2016-11-23

**Authors:** Sanket J. Joshi, Yahya M. Al-Wahaibi, Saif N. Al-Bahry, Abdulkadir E. Elshafie, Ali S. Al-Bemani, Asma Al-Bahri, Musallam S. Al-Mandhari

**Affiliations:** ^1^Central Analytical and Applied Research Unit, College of Science, Sultan Qaboos UniversityMuscat, Oman; ^2^Department of Petroleum and Chemical Engineering, College of Engineering, Sultan Qaboos UniversityMuscat, Oman; ^3^Department of Biology, College of Science, Sultan Qaboos UniversityMuscat, Oman; ^4^Petroleum Development OmanMuscat, Oman

**Keywords:** *Bacillus licheniformis*, surface tension, interfacial tension, lichenysin, wettability alteration, core flood, microbial enhanced oil recovery

## Abstract

The biosurfactant production by *Bacillus licheniformis* W16 and evaluation of biosurfactant based enhanced oil recovery (EOR) using core-flood under reservoir conditions were investigated. Previously reported nine different production media were screened for biosurfactant production, and two were further optimized with different carbon sources (glucose, sucrose, starch, cane molasses, or date molasses), as well as the strain was screened for biosurfactant production during the growth in different media. The biosurfactant reduced the surface tension and interfacial tension to 24.33 ± 0.57 mN m^−1^ and 2.47 ± 0.32 mN m^−1^ respectively within 72 h, at 40°C, and also altered the wettability of a hydrophobic surface by changing the contact angle from 55.67 ± 1.6 to 19.54°± 0.96°. The critical micelle dilution values of 4X were observed. The biosurfactants were characterized by different analytical techniques and identified as lipopeptide, similar to lichenysin-A. The biosurfactant was stable over wide range of extreme environmental conditions. The core flood experiments showed that the biosurfactant was able to enhance the oil recovery by 24–26% over residual oil saturation (S_or_). The results highlight the potential application of lipopeptide biosurfactant in wettability alteration and microbial EOR processes.

## Introduction

Recent updates in our understanding of the microbial metabolisms and their biochemical pathways lead to different applications in petroleum industries like enhancing oil recovery, biodegradation of oil-based waste, treatment of oil-field produced water, bioremediation of oil-spills, etc. At present the worldwide crude oil market is quite volatile with fluctuations in prices. Still, there is an urgent need for new, efficient and superior oil recovery technologies, known as enhanced oil recovery (EOR) techniques. Amongst several types of EOR technologies, microbial enhanced oil recovery (MEOR) is considered to be quite promising one, environmental friendly green-technology (Belyaev et al., 2004; Sen, 2008; Al-Sulaimani et al., 2011b; Zahner et al., 2011). Chemical surfactants and polymers are amongst highly utilized compounds in petroleum industries during EOR applications, for reducing surface tension (ST), interfacial tension (IFT), for generating oil-in water OR water-in-oil emulsions, heavy oil recovery by acting as a viscosifying agent, etc. However, recently the trend has been shifted toward applications of green-compounds or bio-products, mainly because of the environmental concerns. Biosurfactants are such biologically produced surfactants by microorganisms, plants, and animals. Biosurfactants are reported for reducing ST and IFT between oil/water/rock interface, altering the rock wettability, initiating oil/water emulsions, or changing the hydrophilic–lipophilic balance (HLB), thus enhancing the oil recovery (Desai and Banat, 1997; Sen, 2008; Banat et al., 2010). Biosurfactants have been the focus of extensive research because of several advantages over chemical surfactants: considerably lower toxicity, better biodegradability and synthesis from cheaper-agro-industrial waste or renewable raw materials (Marchant and Banat, 2012a,b). Several companies around the globe (UK, USA, Japan, Germany, China) are producing different types of biosurfactant (such as rhamnolipids, lipopeptides, sophorolipids, etc.), with focus on different applications (such as pharmaceuticals, cosmetics, antimicrobials and anticancer, bioremediation, EOR, etc.) (Sekon and Rahman, 2014). Even with all these new developments and increased interest by prominent business-houses, the economically availability and wide-spread applications of biosurfactants are still limited in petroleum industries. The major reason is the availability of comparatively cheaper chemical surfactants and higher cost of biosurfactant production. Several researchers have reported different ways to tackle higher production costs by applying statistical optimization methods or by use of cheaper agro-industrial waste products (Sen and Swaminathan, 2004; Joshi et al., 2007, 2008a; Makkar et al., 2011; Al-Bahry et al., 2013; Saimmai et al., 2013; Chooklin et al., 2014). Different types of biosurfactants are reported, amongst which lipopeptide type of biosurfactant produced by *Bacillus* group are widely studied. Surfactins and lichenysins produced by *Bacillus subtilis* and *Bacillus licheniformis* strains are reported for their high surface activities and other beneficial properties suitable for several applications like enhancing oil recovery (Jean-Marc et al., 2003).

Sultanate of Oman is oil producing Middle Eastern country, where various EOR technologies are employed to enhance the crude oil recovery from the declining reservoirs in the sultanate. To check the potential of biosurfactant based MEOR applications in Oman, we isolated and screened several spore forming bacteria and analyzed for biosurfactant production (Al-Sulaimani et al., 2011a). Amongst several isolates, *B. licheniformis* W16 showed biosurfactant production and was further selected. The biosurfactant production by this isolate was studied in nine different reported minimal media, and five different carbohydrates were tested to find suitable carbon source in selected better media. The biosurfactant was studied further for wettability alteration (contact angle determination), critical micelle dilution (CMD), extracted and characterized, stability under harsh conditions and MEOR studies by core-flood using Berea sandstone core-plugs under reservoir conditions.

## Materials and Methods

### Chemicals and Reagents

All chemicals, reagents and hydrocarbon (*n*-heptane and *n*-hexadecane) were Analytical Reagent (AR) grade or HPLC grade, purchased from Sigma-Aldrich, Co., USA. Cane molasses and date molasses were purchased from the local market, Oman. Formation water and light crude oil was kindly provided by Petroleum Development Oman (PDO), Oman.

### Microorganism

Previously reported bacterial isolate *B. licheniformis* W16 (GenBank Accession number GU945226), isolated from soil near an Omani oil well (Al-Sulaimani et al., 2011a) was used for all experiments. The isolate was maintained aerobically on Luria-Bartani (LB) agar plates and was regularly transferred into fresh LB medium for short-term storage. Stock cultures of pure isolate were prepared in 40% glycerol and stored below −80°C, for long-term preservation.

### Screening of Biosurfactant Production Media

For biosurfactant production studies, LB broth was used as a seed medium. After 15 h incubation at 40°C (OD_660__nm_-1.0), 2% (v/v) seed culture was transferred to 50 ml each of different minimal production media in 250 ml Erlenmeyer flasks. For initial screening, seven different minimal media and two media based on only molasses, for biosurfactant production were used for current studies, as reported by Al-Sulaimani et al. (2011a). The compositions of minimal media (M) tested with different carbohydrates as a carbon sources are as follows (g per 1000 ml distilled water): **M1** (Joshi et al., 2008b) – Glucose, 34; NH_4_NO_3_, 1.0; KH_2_PO_4_, 6.0; Na_2_HPO_4_, 2.7; MgSO_4_.7H_2_O, 0.1; FeSO_4_.7H_2_O, 0.00165; MnSO_4_.4H_2_O, 0.001; CaCl_2_, 0.0012; Na_2_-EDTA, 0.000745. **M2** (Joshi et al., 2007) – Glucose, 11.0; NaNO_3_, 4.4; MgSO_4_.7H_2_O, 0.8; KCl, 0.4; CaCl_2_, 0.27; H_3_PO_4_ (85.4%), 1.0 ml; Trace elements, 10 ml l^−1^. **M3A** (Joshi et al., 2008a) – Cane molasses, 80.0. **M3B** – Date molasses, 80.0. **M4** (Landy et al., 1948) – Glucose, 20.0; Sodium glutamate, 5.0; KH_2_PO_4_, 1.0; MgSO_4_.7H_2_O, 0.5; FeSO_4_.7H_2_O, 0.15; MnSO_4_.4H_2_O, 0.005; CuSO_4_, 0.16; Yeast Extract, 1.0. **M5** (Jenny et al., 1991) – Glucose, 20.0; NaNO_3_, 4.0; MgSO_4_.7H_2_O, 0.4; Na_2_-EDTA, 0.2; H_3_PO_4_ (85.4%), 0.5 ml; Trace elements, 1 ml l^−1^. **M6** (Youssef et al., 2007) – Sucrose, 10.0; K_2_HPO_4_, 13.9; KH_2_PO_4_, 2.7; MgSO_4_.7H_2_O, 0.25; Yeast Extract, 1.0; NaCl, 50.0; (NH_4_)_2_SO_4,_ 1.0; Trace elements, 10 ml l^−1^. **M7** (Cooper et al., 1981) – Glucose, 20.0; NH_4_NO_3_, 4.0; KH_2_PO_4,_ 4.08; Na_2_HPO_4_, 7.12; MgSO_4_.7H_2_O, 0.2; FeSO_4_.7H_2_O, 0.0011; MnSO_4_.4H_2_O, 0.00067; CaCl_2_, 0.00077; Na_2_-EDTA, 0.00148. **M8** (Mukherjee et al., 2009a) – Sucrose, 20.0; NH_4_NO_3,_ 3.3; K_2_HPO_4,_ 2.2; KH_2_PO_4_, 0.14; MgSO_4_.7H_2_O, 0.6; FeSO_4_.7H_2_O, 0.2; CaCl_2_, 0.04; NaCl, 0.01; Trace elements, 0.5 ml l^−1^. The flasks were incubated in a temperature controlled incubator shaker at 40°C, 160 rpm. The carbon sources were filter sterilized and added separately to minimal media after autoclaving. Samples were withdrawn at every 24 h interval and analyzed for pH, growth (OD_660_), biopolymer production (viscosity), and biosurfactant production –ST and IFT.

### Effect of Different Carbon Sources on Biosurfactant Production

To check the effect of different carbon sources, five different carbohydrates were substituted to glucose and sucrose, in two selected minimal production media composition – M7 and M8. Seed medium (LB broth) was inoculated with loopful of bacteria and after 15 h incubation at 40°C (OD_660__nm_-1.0), 2% (v/v) was transferred to production media – M7 and M8 (50 ml in 250 ml Erlenmeyer flasks) containing different carbohydrates (glucose, sucrose, starch, cane molasses, or date molasses) at 2% (w/v) concentrations. The flasks were incubated in a temperature controlled incubator shaker at 40°C, 160 rpm for up to 72 h. The carbon sources were filter sterilized and added separately to minimal media after autoclaving. Samples were withdrawn at every 24 h interval and analyzed for growth (OD_660_), and biosurfactant production – ST and IFT. All measurements were made on cell-free broth after centrifugation (12,096* × g* for 20 min) and analyzed at room temperatures. The experiments were performed in duplicate and the reported results are the mean of three independent experiments with standard deviation (SD) values.

### Biopolymer Analysis

The samples were analyzed for biopolymer production using viscosity measurements at every 24 h interval up to 72 h. One ml each of different cell-free samples was analyzed for any increase in viscosity using Quarzviskosimeter (QVis 01/L, Flucon, F5 – Technologie, Germany) at room temperatures (25 ± 2.0°C).

### Biosurfactant Analysis, Contact Angle Measurements, and Critical Micelle Dilution (CMD) Measurements

Biosurfactant production was analyzed by periodic measurements of any changes in surface activity – ST and IFT of cell-free samples by ‘pendant drop method’ using the Drop Shape Analyzing system – DSA 100 (KRÜSS, Germany). The IFT measurements were done against *n*-heptane or *n*-hexadecane. Contact angles of the abiotic controls (un-inoculated media) and biosurfactants were measured using the Drop Shape Analysis System, DSA100 (KRÜSS, Germany), as reported by Al-Sulaimani et al. (2012). All measurements were done in triplicates at ambient temperature (25 ± 2.0°C) and atmospheric pressure (1 atm) and the average values were reported. The CMD was estimated by measuring the ST and IFT at varying dilutions of the sample (Joshi et al., 2008a).

### Biosurfactant Extraction and Characterization

The biosurfactant was partially purified and extracted by the acid precipitation method (Youssef et al., 2005). Bacterial cells were separated by centrifuging at 12,096* × g* for 20 min at 20°C (Beckman, JLA 16.250 rotor, USA), and the pH of the cell-free broth was adjusted to 2.0 using 6 M HCl. This acidified cell-free solution was incubated overnight at 4°C, and the precipitated biosurfactant was collected by centrifuging the solution at 12,096* × g* for 25 min at 4°C. The collected biosurfactant pellet was dissolved in 100 ml distilled water, and its pH was adjusted to 8.0, with 1 N NaOH. Crude biosurfactant powder was collected by spray-drying reconstituted biosurfactant solution using Mini-Spray Dryer B-290 (BÜCHI, Switzerland), at set temperature program ranging between 100 and 160°C. Extracted crude biosurfactant was then tested for its stability under different conditions, chemical characterization and in core-flooding experiments.

#### Fourier Transform Infrared Spectroscopy (FTIR)

The structural groups of the biosurfactant were partially identified using Fourier transform infrared spectroscopy (FTIR). Infrared (IR) absorption spectra were obtained with a Perkin–Elmer grating 1000 IR (Norwalk, CT, USA) in a dry atmosphere at Sultan Qaboos University, Oman. For analysis, 1 mg of crude biosurfactant was mixed with 100 mg of KBr and pressed for 30 s, to obtain translucent pellets, and FTIR spectra were collected between 400 and 4000 wave numbers (cm^−1^)_._

#### High Performance Thin Layer Chromatography–Electrospray Ionization Mass Spectroscopy (HPTLC–ESI–MS)

Biosurfactant was separated in a completely automated HPTLC system (CAMAG, Switzerland), extracted by TLC–MS interface and were analyzed directly by ESI–MS at Central Analytical and Applied Research Unit (CAARU), Sultan Qaboos University, Oman. Twenty microliter of biosurfactant (0.1 g l^−1^) sample was spotted onto a HPTLC plate (10 cm × 10 cm), Silica gel 60 – F_254_ (Merck, Germany). The samples were spotted by automatic TLC sampler 4 (ATS 4) spotting device (CAMAG, Switzerland), using nitrogen gas. Four different solvent systems (SS) were checked for better separation of biosurfactant components: SS1: Chloroform: Methanol: Ammonium Hydroxide (65:25:4); SS2: Chloroform: Methanol: Acetone: Acetic acid (90:10:6:1); SS3: Chloroform: Methanol: Water (65:25:4); and SS4: Butanol: Acetic acid: Water (4:1:1). The HPTLC plates were separately developed using above mentioned four different solvent systems in an automatic developing chamber ADC 2 (CAMAG, Switzerland) with remote operation from winCATS software. The HPTLC plates were evaluated by TLC visualizer (CAMAG) under direct Ultra Violet (UV 254 nm) light, and images were captured. The separated biosurfactant fractions were also qualitatively detected and compared by TLC scanner 4 (CAMAG, Switzerland) under UV light (254 nm), extracted and eluted by TLC–MS interface (CAMAG, Switzerland). The TLC–MS interface head (oval, 4 mm × 2 mm) was connected to the pump (11 PLUS, HARVARD APPARATUS, Holliston, MA, USA) and the extraction was performed at a flow rate of 10 μl/min, with methanol: acetonitrile (50% diluted with water) – 1:10. The interface outlet was directly connected with the ESI–MS (Qaattro Ultima^TM^ Pt, Micromass^®^, UK). The experimental conditions were: capillary voltage, 3.0 kV; cone voltage, 35 V; lens voltage, 0.0 V; source block temperature, 100°C; desolvation temperature, 120°C; analyzed under both positive and negative modes in the m/z range of 900–1200 Da, using Mass Lynx (V4.0) software.

#### Matrix Assisted Laser Desorption Ionization Time-of-Flight Mass Spectrometry Analysis (MALDI TOF–MS)

The MALDI-TOF analysis was performed at CAARU, Sultan Qaboos University, on UltraFlextreme (Bruker Daltonics, Bremen, Germany) operating in positive reflectron mode in the m/z range of 50–2000 Da. Two μl of 2, 5- Dihydroxy benzoic acid (DHB) matrix (20 mg/ml) in TA 30 (30:70 v/v ACN:TFA 0.1%TFA) was premixed with 2 μl of the sample solution. 1 μl of the sample-mixture was applied to the steel target plate, and air-dried at room temperature. The spectra were acquired using FlexControl software (v3.3), and FlexAnalysis Software (v3.3, Bruker Daltonics, Bremen, Germany) was used for visualization and initial data processing, as previously reported (Elshafie et al., 2015).

#### Nuclear Magnetic Resonance (NMR) Analysis

The nuclear magnetic resonance (NMR) analysis were performed at room temperature in Bruker Avance 400 MHz spectrometer equipped with 5 mm BBO probe at Department of Chemistry, Sultan Qaboos University. The proton (^1^H) NMR experiment was run using zg30 pulse program operating at 400.13 MHz. Acquisition parameter were as follows: 90° proton pulse width of 14.80 μs, relaxation delay of 2 s, 2048 scans. The proton decoupled ^13^C NMR experiments were carried out using composite pulse decoupling scheme operating at 100.61 MHz. Acquisition parameter were as follows: 90° proton pulse width of 9.80 μs, relaxation delay of 2 s, 9216 scans. The Spectra were recorded in CD_3_OD at 296.2 K and processed using XWIN 3.5 software.

### Biosurfactant Stability Studies

Biosurfactant stability was studied at wide range of temperatures (40–100°C), pH (2.0–12.0) and different salinities (2–15% v/v). All control experiments were conducted at room temperature, pH 7.0 and 0% salt concentration. For the temperature stability test, the cell-free samples were filled in 10 ml serum bottles, sealed with butyl rubber stoppers and aluminum crimps, and incubated at respective temperatures, to avoid any loss due to evaporation. The biosurfactant broth was also subjected to autoclave conditions (121°C, 15 psi for 30 min) in order to investigate the effect of such environment on the surface activity. To check the effect of salt concentrations, different concentrations of NaCl were added to cell-free biosurfactant broth, dissolved completely and incubated at room temperature. The stability of the biosurfactant was also determined by measuring the ST and IFT at different pH values (by addition of 6N HCl or 1N NaOH) at room temperature.

### Berea Sandstone Core-Plugs and Fluid Samples

A set of Berea sandstone cores (1.5 inch diameter* ×* 3 inch long) with average porosity and permeability of 18–22% and 250–260 mD respectively, were used for the core-flood experiments. The formation water and crude oil used in these experiments were provided by Petroleum Development Oman. Formation water was filtered prior to use, by Millipore Filtration Unit (0.45 μm). The crude oil used for core-flood experiments was light oil with API 36.51° and 1.77cp viscosity. The formation water was analyzed for anions, cations, and different parameters. Multi-parameter equipment (Multi 9310, WTW, Germany) was used for: conductivity, resistivity, total dissolved solids (TDS), salinity, temperature and pH meter (Jenway 3505, UK) was used for analyzing the pH; Anton Paar Density meter (DMA 4500M) was used for measuring specific gravity, as per manufacturer instructions. Anions and major cations analysis was done using Ion chromatography (850 Professional IC AnCat MCS and 858 Professional Auto sampler, Metröhm, Switzerland). For anion analysis Metrosep A Supp 7–250 (250 mm *×* 4.0 mm) column was used and analyzed by MagIc Net^TM^ software. The running conditions were: Mobile phase – 3.6 mM Sodium carbonate; Regenerant – 200 mM Sulfuric acid; Rinsing solution – Ultrapure water; Temperature – 45°C; Flow rate – 0.7 ml/min. For cation analysis Metrosep C 4 – 150 (150 mm× 4.0 mm) column was used, and analyzed by MagIc Net^TM^ software. The running conditions were: Mobile phase – 1.7 mM HNO_3_ and 0.7 mM DPA; Rinsing solution – Ultrapure water; Temperature – 45°C; Flow rate – 0.9 ml/min. Remaining metal cation analysis was performed using – Inductively coupled plasma – mass spectrometer (ICP-MS, Bruker aurora M90); Mode –Aqueous Analysis; Ar Plasma Flow- 16.5 Lit Pm, Aux- 1.65 Lit Pm, Sheath- 0.2 Lit Pm, Neb- 1.0 Lit Pm.

### Core-Flood Experiments

Prior to core-flooding experiments, all core-plugs were thoroughly cleaned by chloroform and methanol (75:25) in the Soxhlet apparatus (Al-Sulaimani et al., 2011a). After cleaning, the core-plugs were dried in a hot air oven at 65°C for 24 h. The cleaned and dried core-plugs were then saturated with filtered formation brine using vacuum desiccators for 24 h to measure the pore volume (PV), using the dry and wet weights of the core. The core-plugs were then flooded with crude oil at 24 cm^3^ h^−1^ until no more brine was produced. The oil initially in place (OIIP) was determined by the volume of brine displaced with crude oil. Then, cores were brine-flooded (24 cm^3^ h^−1^) until no further oil was produced, and the residual oil was calculated by measuring the amount of oil produced from the brine-flood. Then, 5 PV of the cell-free biosurfactant broth was injected as a tertiary recovery stage and extra oil recovery was determined. All core-flood experiments were conducted at 60°C to mimic the average reservoir temperature of the field of interest.

## Results and Discussion

Microbial enhanced oil recovery is regarded as one among the most promising green-technologies that can be potentially implemented with an exceptionally low operating cost. Several of the bacterial products are reported for playing a role in MEOR, where biopolymers and biosurfactants are reported as key players (Belyaev et al., 2004; Sen, 2008; Marchant and Banat, 2012a). Amongst different kind of biosurfactants, low molecular compounds – lipopeptides produced by spore-forming bacteria are regarded as having potential role in MEOR. Several morphologically different spore-forming bacteria were isolated from different oil contaminated soil samples collected from Oman, and screened for biosurfactant production. Out of those isolates, a facultative aerobic bacteria *B. licheniformis* W16 produced biosurfactant (Al-Sulaimani et al., 2011a) and propagated under anaerobic conditions in the fractured carbonate rocks and enhanced the oil recovery by selective plugging (Al-Hattali et al., 2013). Thus, it was further selected in current study to investigate the effect of different carbohydrates in the minimal media and the potential of produced biosurfactant in biosurfactant based MEOR. The growth and production profile in the nine different production media are shown in **Table 1**. The better growth was observed in media 2, 5, and 8 within 24 h, whereas very little growth was observed in media 4. One possible reason for little growth in media 4 could be because of the acidic pH after 24 h, as compared to observed neutral to alkaline pH in rest of the media (**Table 1**), and this strain showed better growth in neutral to alkaline range (data not shown). The lowest ST and IFT values were observed in media 7 and 8, as 25.89–38.88 mN m^−1^ and 4.48–18.88 mN m^−1^ respectively. Therefore, media 7 (M7) and 8 (M8) were further selected for carbon source optimization studies. None of the media showed much increase in the viscosities even after 72 h, and the viscosities varied between 1.3 and 1.5 mPa s, for both abiotic controls and experimental samples. The viscosities of some of the biopolymers are reported to be in the range of 43–21535 cP (Joshi et al., 2016). Hence, it was concluded that this isolate *B. licheniformis* W16 produced only biosurfactant and not biopolymer, under tested environmental conditions in these nine minimal media.

**Table 1:** The growth (OD_660_), biosurfactant production (ST and IFT), biopolymer production (viscosity) and pH profile of *Bacillus licheniformis* W16 in carbohydrates based minimal production media.

		Minimal production medium								
Test parameters	Time (h)	M1	M2	M3A	M3B	M4	M5	M6	M7	M8
Growth (OD_660_)	0	0.0 ± 0.0	0.0 ± 0.0	0.39 ± 0.08	0.30 ± 0.09	0.15 ± 0.05	0.0 ± 0.0	0.0 ± 0.0	0.0 ± 0.0	0.05 ± 0.02
	24	1.651 ± 0.15	2.011 ± 0.18	0.889 ± 0.08	1.005 ± 0.10	0.329 ± 0.05	2.11 ± 0.08	1.365 ± 0.05	1.585 ± 0.15	1.945 ± 0.18
	48	1.633 ± 0.25	2.239 ± 0.05	1.25 ± 0.16	1.129 ± 0.18	0.334 ± 0.04	2.351 ± 0.06	1.364 ± 0.08	1.762 ± 0.10	2.145 ± 0.16
	72	1.581 ± 0.16	2.339 ± 0.21	1.311 ± 0.09	1.223 ± 0.10	0.483 ± 0.08	2.412 ± 0.08	1.501 ± 0.05	1.731 ± 0.08	2.109 ± 0.10
ST (mN m^−1^)	0	71.30 ± 0.56	72.00 ± 0.60	63.56 ± 1.12	62.11 ± 1.30	71.20 ± 0.68	72.60 ± 0.50	68.76 ± 0.66	71.08 ± 0.45	73.12 ± 0.25
	24	62.79 ± 0.89	57.43 ± 1.10	67.10 ± 1.00	67.56 ± 1.40	70.78 ± 0.80	52.29 ± 0.80	66.22 ± 0.58	64.50 ± 1.00	65.39 ± 0.95
	48	61.82 ± 1.10	61.05 ± 1.08	64.74 ± 1.06	60.93 ± 1.20	70.98 ± 0.56	57.83 ± 0.66	66.19 ± 0.92	38.88 ± 0.86	25.89 ± 0.44
	72	61.64 ± 1.06	37.09 ± 0.88	64.60 ± 0.94	64.81 ± 1.28	69.74 ± 0.55	26.82 ± 0.56	65.32 ± 1.00	30.35 ± 0.80	25.27 ± 0.26
IFT^∗^ (mN m^−1^)	0	47.32 ± 0.60	46.96 ± 0.55	30.56 ± 0.46	29.86 ± 0.40	41.16 ± 0.52	47.35 ± 0.46	38.49 ± 0.44	46.56 ± 0.25	48.00 ± 0.54
	24	25.46 ± 0.30	27.22 ± 0.25	32.54 ± 0.52	31.38 ± 0.50	37.95 ± 0.25	31.12 ± 0.20	33.53 ± 0.55	28.95 ± 0.62	24.76 ± 0.40
	48	30.58 ± 0.56	25.85 ± 0.45	30.82 ± 0.40	29.82 ± 0.42	38.11 ± 0.20	28.75 ± 0.40	30.92 ± 0.64	18.18 ± 0.42	4.48 ± 0.62
	72	29.88 ± 0.45	23.73 ± 0.40	30.53 ± 0.24	31.09 ± 0.50	37.71 ± 0.40	19.10 ± 0.35	30.33 ± 0.80	14.83 ± 0.24	8.27 ± 0.44
Viscosity (mPa.s)	0	1.30 ± 0.08	1.09 ± 0.05	1.33 ± 0.04	1.32 ± 0.02	1.18 ± 0.05	1.21 ± 0.02	1.26 ± 0.05	1.15 ± 0.04	1.14 ± 0.06
	24	1.32 ± 0.08	1.30 ± 0.06	1.32 ± 0.05	1.39 ± 0.06	1.19 ± 0.05	1.29 ± 0.04	1.36 ± 0.05	1.39 ± 0.02	1.30 ± 0.02
	48	1.41 ± 0.06	1.26 ± 0.02	1.39 ± 0.06	1.48 ± 0.08	1.19 ± 0.07	1.40 ± 0.06	1.13 ± 0.01	1.40 ± 0.04	1.29 ± 0.05
	72	1.39 ± 0.06	1.19 ± 0.04	1.46 ± 0.02	1.42 ± 0.08	1.23 ± 0.02	1.50 ± 0.02	1.05 ± 0.02	1.36 ± 0.06	1.25 ± 0.03
pH	0	7.16 ± 0.10	7.02 ± 0.12	7.3 ± 0.08	7.5 ± 0.11	7.22 ± 0.08	7.34 ± 0.12	7.05 ± 0.05	7.27 ± 0.04	7.37 ± 0.02
	24	7.02 ± 0.08	7.48 ± 0.12	6.66 ± 0.52	6.95 ± 0.25	5.03 ± 0.52	8.06 ± 0.55	5.71 ± 0.52	7.94 ± 0.25	7.40 ± 0.48
	48	7.13 ± 0.08	8.19 ± 0.80	6.75 ± 0.45	7.15 ± 0.28	5.05 ± 0.40	8.3 ± 0.68	6.43 ± 0.25	7.75 ± 0.20	9.51 ± 0.20
	72	7.02 ± 0.06	10.13 ± 0.06	6.52 ± 0.40	7.21 ± 0.08	4.75 ± 0.25	10.25 ± 0.20	6.51 ± 0.43	9.48 ± 0.52	10.02 ± 0.15

^∗^*IFT measured against n-heptane*.

**FIGURE 1:**
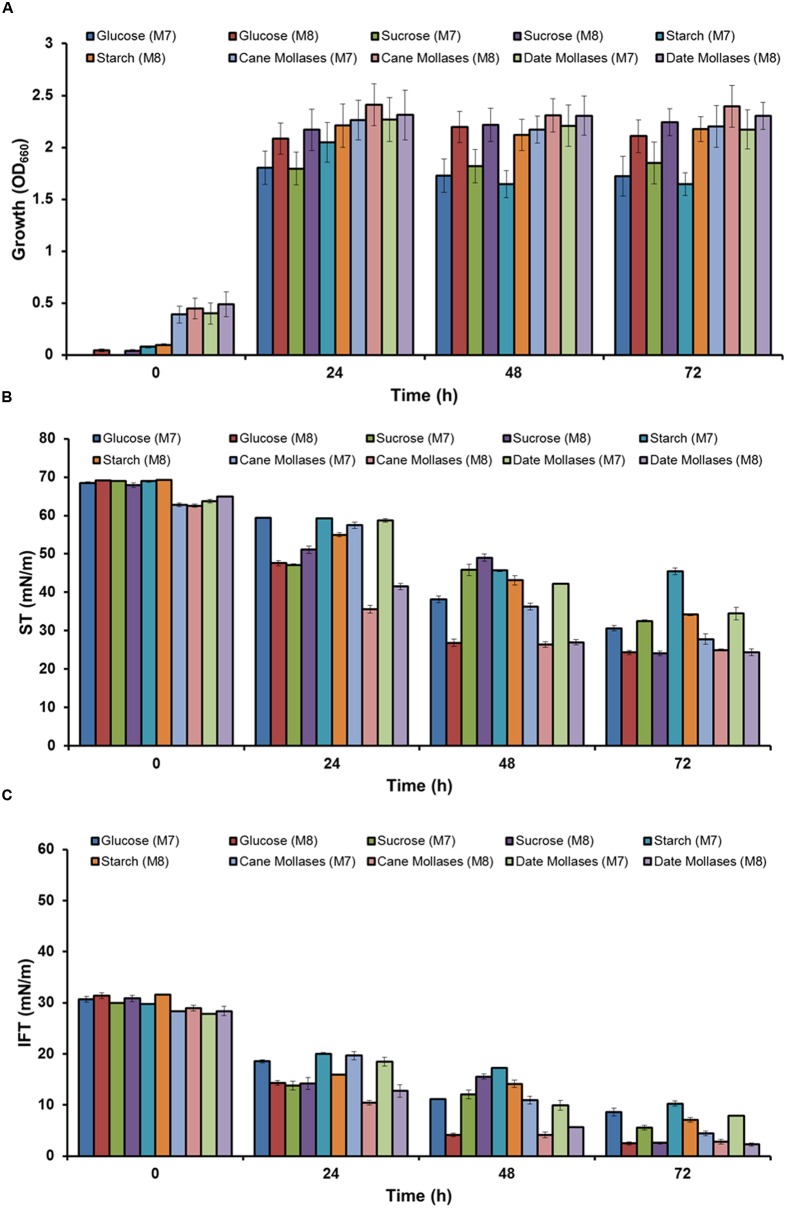
**The growth (A)** and biosurfactant production profile –ST **(B)** and IFT **(C)** (against hexadecane), of *Bacillus licheniformis* W16 in in two minimal production media – M7 and M8 with five different carbohydrates (glucose, sucrose, starch, cane molasses, or date molasses) as a carbon source.

As media M7 and M8 showed better growth and biosurfactant production, both media were selected for further screening of different types of carbohydrates as a carbon sources – glucose, sucrose, starch, cane moleasses, or date molasses. Better growth was observed in M8 media as compared to M7, in all five carbon sources, and highest growth (OD_660_ – 2.3–2.4) was observed in M8 media containing cane or date molasses (**Figure 1A**). It was observed that in all media ST was reduced to <35 mN m^−1^, from 62 to 70 mN m^−1^, within 72 h (**Figure 1B**), except in starch containing Media M7, where higher ST was observed (45.42 ± 0.88 mN m^−1^). Similar trend was observed for IFT also, where starch containing media M7 had higher IFT (10.27 ± 0.52) as compared to all other conditions (**Figure 1C**). The lower ST and IFT were observed in M8 media containing glucose, cane molasses or date molasses, and glucose containing media showed the lowest ST and IFT values of 24.33 ± 0.31 mN m^−1^ and 2.47 ± 0.32 mN m^−1^ respectively. These observed values of ST and IFT are in accordance with several other reports by researchers for biosurfactant production by *Bacillus* species (Joshi et al., 2008a; Al-Bahry et al., 2013; Chandankere et al., 2013; Pereira et al., 2013). In this study, it was observed that isolate *B. licheniformis* W16 produced a potent biosurfactant using either glucose or cane molasses as a carbon source. Glucose based media M8 was used for biosurfactant production and further studies, as it gave better results in ST and IFT reduction.

**FIGURE 2:**
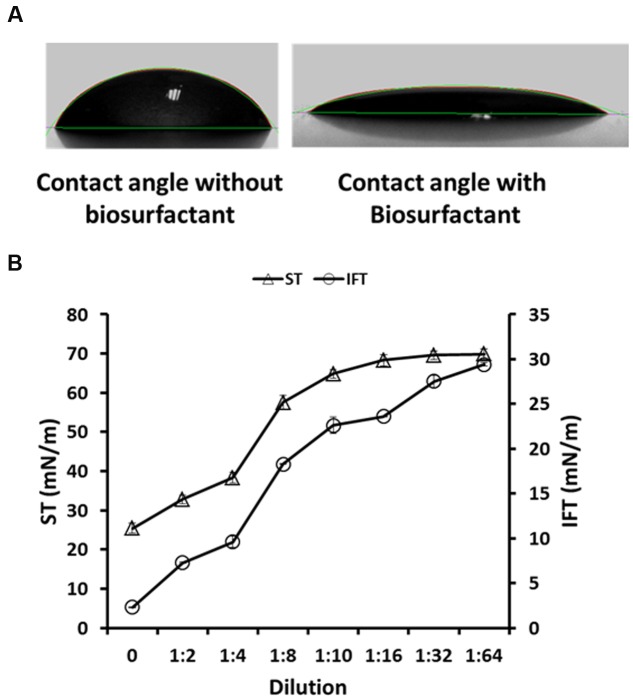
**The contact angle of abiotic control medium – without biosurfactant (55.67 ± 1.6°), and W16 biosurfactant (19.54 ± 0.96°), on a hydrophobic surface (A)** and the CMD determination for W16 biosurfactant **(B)**.

Any alteration at the reservoir ‘oil-water-rock’ interface, leads to change in the surface-wettability properties (oil-wet to water-wet and vice versa), which has also been proposed as one of the mechanism responsible for EOR (Kowalewski et al., 2006; Al-Sulaimani et al., 2012; Al-Wahaibi et al., 2014). The *B. licheniformis* W16 biosurfactant produced from glucose containing M8 medium, was studied for any effect on changes in the contact angle on a hydrophobic surface (provided with the instrument – DSA 100, KRÜSS, Germany). The contact angle was reduced from 55.67 ± 1.6° of un-inoculated abiotic control media to 19.54 ± 0.96° in cell-free biosurfactant broth (**Figure 2A**). Karimi et al. (2012) studied the effect of microbial solutions (using an *Enterobacter cloacae* strain) on 7–21 days aged glass surfaces, and have reported that it alters the wettability of hydrophobic glass surfaces toward more water-wet conditions. Al-Sulaimani et al. (2012) reported that biosurfactant produced by *B. subtilis* W19 changed the contact angle of distilled water from 70.6 ± 0.3° to 25.32 ± 0.06° at 0.25% (w/v) biosurfactant. Al-Wahaibi et al. (2014) also reported changes in wettability of hydrophobic surface from 58.7 ± 0.85° to 28.4 ± 1.03° and 27.2 ± 0.72° by biosurfactant produced by *B. subtilis* B30 in glucose or molasses based minimal media. In the current study, we observed that biosurfactant changed the wettability of hydrophobic surface toward more water-wet, which is beneficial during EOR applications. To the best of our knowledge this is the first report of wettability alteration using biosurfactant produced by *B. licheniformis* strain. Along with reduction in ST and IFT, wettability alteration by W16 biosurfactant could also play an important role in improving oil recovery at field scale applications. It is reported that the dilution at which the ST/IFT begins to increase is termed the CMD, which is actually the factor by which the effective biosurfactant concentration exceeds the critical micelle concentration (Ghurye et al., 1994; Al-Wahaibi et al., 2014). The ST and IFT values increased sharply after 4X dilution, where the ST and IFT values observed were 38.28 ± 1.36 mN m^−1^ and 9.61 ± 0.65 mN m^−1^ respectively (**Figure 2B**). Therefore, the observed CMD values for W16 biosurfactant was 4X. Makkar and Cameotra (1997) have reported the biosurfactant production by two *B. subtilis* strains which reduced the ST in the range of 30–40 dynes cm^−1^ with 10 X–100 X CMD values after 96 h. Joshi and Desai (2013) reported CMD values of 75X–100X for biosurfactants mixtures produced by different *Bacilli* strains in carbohydrate based minimal media. Al-Wahaibi et al. (2014) reported CMD values of 8X for biosurfactant produced by *B. subtilis* B30. The CMD values observed for W16 biosurfactant was comparatively lower than biosurfactants produced by other *Bacilli* strains.

**FIGURE 3:**
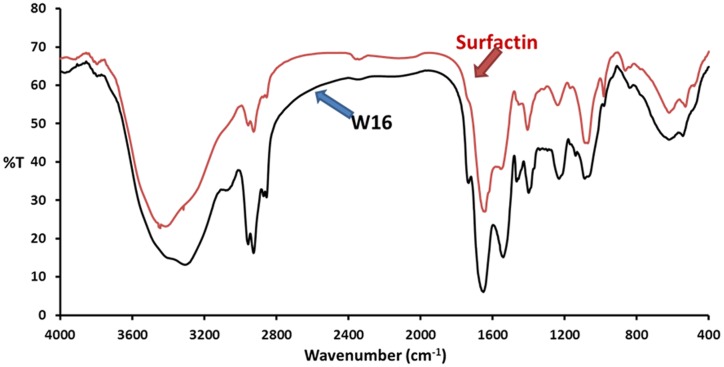
**The FTIR absorption spectra of biosurfactant produced by *B. licheniformis* W16, and reference standard lipopeptide biosurfactant – surfactin (Sigma Chemicals, USA)**.

To determine the yield of biosurfactant, it was extracted by acid-precipitation and crude-powder was collected by spray-drying. The yield of partially purified biosurfactant was 0.52 ± 0.1 g l^−1^, which was used for structural identification by FTIR. The IR spectra of biosurfactants produced by *B. licheniformis* W16 is shown in **Figure 3**. The IR spectrum in KBr showed bands characteristic of peptides at 3300–3400 cm^−1^ (N-H stretching mode) and at 1650–1700 cm^−1^ (stretching mode of the CO–N bond). The bands at 1200–1400 cm^−1^ reflected the aliphatic chains (–CH_3_, –CH_2_–) of the isolated fraction (Al-Sulaimani et al., 2011a; Al-Wahaibi et al., 2014; Joshi et al., 2015). These results imply the presence of aliphatic groups as well as a peptide-like moiety in the biosurfactant. For comparison of the biosurfactant, surfactin (Sigma chemicals, USA) was also analyzed by FTIR (**Figure 3**). The IR spectrum of surfactin and W16 biosurfactant was quite similar, which suggested the similarity in the structure of the biosurfactant produced by *B. licheniformis* W16 with lipopeptide surfactin. Structurally, similar lipopeptide biosurfactants – surfactins and lichenysins are reported as excellent surface active agents with potential in EOR applications (Jean-Marc et al., 2003).

**FIGURE 4:**
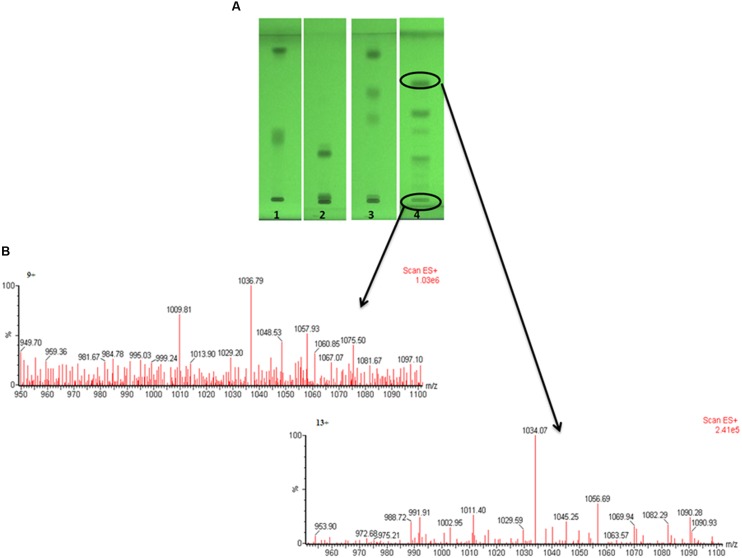
**The HPTLC plate developed with four different solvent systems, showing separated biosurfactant under UV 254 nm (A)**, and the mass spectra of two bands directly extracted by TLC–MS interface and analyzed by ESI–MS **(B)** under positive mode.

Out of four solvent systems used for HPTLC, SS4 gave better separation (**Figure 4A**) as compared to SS1, SS2, and SS3; hence, we used solvent system – SS4 for further characterization of biosurfactant. The TLC or HPTLC are reported as quite useful tool for initial qualitative or quantitative analysis of different types of biosurfactants (Mukherjee et al., 2009b; Ismail et al., 2013; Al-Wahaibi et al., 2014). We further utilized TLC–MS interface for extraction and elution of separated biosurfactant bands and analyzed them directly by ESI – MS under positive and negative modes. Total five major bands were scrapped, eluted and analyzed by ESI–MS. The mass spectra (**Figure 4B**) revealed the major group of peaks at m/z values between 1000 and 1090 (The mass spectra of remaining bands are as Supplementary Figure S1). This group could be attributed to the different variants of surfactins or lichenysins, as previously described (Pereira et al., 2013; Al-Wahaibi et al., 2014; Joshi et al., 2015). The HPTLC–ESI–MS is quite easy and quick technique to identify the biosurfactants. To further confirm the identity of different biosurfactant isoforms, biosurfactant was analyzed using MALDI-TOF-MS.

**FIGURE 5:**
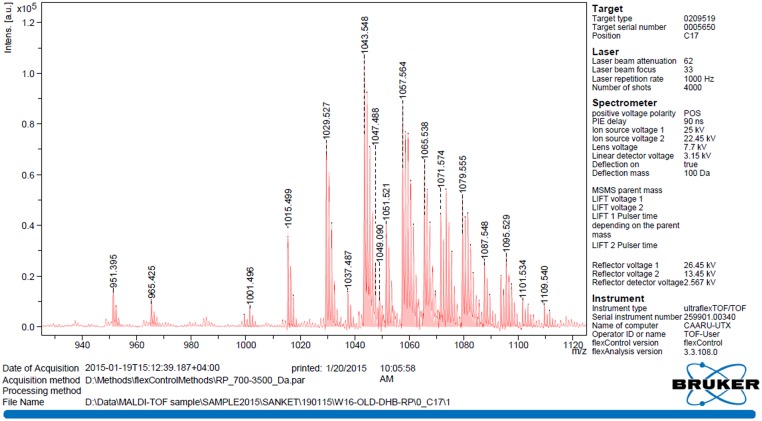
**The MALDI-TOF spectrum of biosurfactant produced by *B. licheniformis* W16**.

MALDI-TOF mass spectrum of the biosurfactant produced by *B. licheniformis* W16 is shown in **Figure 5**. The observed main peaks were similar to the molecular masses of known lipopeptides – lichensyin and their homologs (Horowitz and Griffin, 1991; Mikkola et al., 2000; Li et al., 2008; Nerurkar, 2010; Madslien et al., 2013; Pereira et al., 2013; Zhang et al., 2014). There were a total of 12 main peaks with the m/z value as 1015.5–1087.5. Considering the molecular mass of different homologs of lichenysin (C_12_–C_16_), these peaks were identified as protonated ions [M + H] ^+^ *m/z* 1049.1; sodium adduct ions [M + Na] ^+^ with *m/z* of 1015.5, 1029.5, 1043.5, and 1057.6; sodium adducts [M-H + 2Na]^+^ with *m/z* of 1051.5, 1065.5, and 1079.5; potassium adducts [M + K] ^+^ with *m/z* of 1087.5. MALDI-TOF analysis of W16 biosurfactant showed similarity with lichenysin-A, produced by *B. licheniformis* strains (Grangemard et al., 1999; Mikkola et al., 2000; Li et al., 2008; Zhang et al., 2014; Joshi et al., 2015). They have reported that lichenysin – A is a cyclic heptalipopeptide having a small peptide (Gln, Leu, Leu, Val, Asp, Leu, and Ile) linked to 3-hydroxy fatty acid residue with amide (Gln) and lactone (Ile) bonds forming a cyclic structure, with main fatty acids are 3-hydroxylated tri, tetra, penta, and hexadecanoic acids.

**FIGURE 6:**
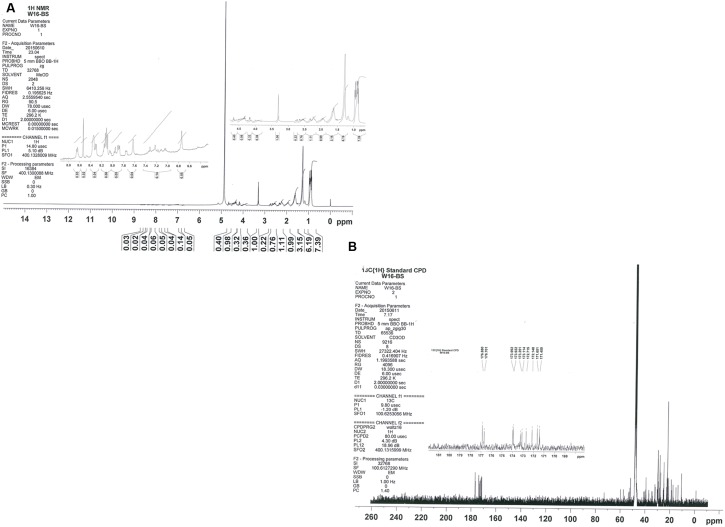
**The NMR spectra for biosurfactant produced by of ***B. licheniformis*** W16 – ^1^H NMR (A)** and ^13^C NMR **(B)**.

The results obtained with ^1^H NMR (**Figure 6A**) further confirmed the lipopeptide nature of the molecule. Seven amide (-NH-) groups were observed in the region from 8.4 to 7.4 ppm, and the alpha-hydrogen (Hαs) of the amino acids showed resonance from 4.7 to 3.8 ppm. A doublet at δ = 0.904 ppm was observed, which indicated a terminal branching in the fatty acyl chain [-(CH_3_)_2_-CH-]. Other multiplets in the upfield region arise as a result of the side chain protons of the amino acids, and remaining spectra confirmed the presence of β-hydroxy fatty acid. The ^13^C-NMR spectrum showed strong signals at 10.744–17.527, 18.638–59.817, and 171.450–176.880 ppm from methyl, methylene, and carboxyl group, respectively (**Figure 6B**). Therefore, the biosurfactant produced by *B. licheniformis* W16 was characterized as lipopeptide, similar to lichenysin-A (Horowitz and Griffin, 1991; Lin et al., 1994; Makkar and Cameotra, 1999; Saimmai et al., 2013; Zhang et al., 2014; Joshi et al., 2015).

**FIGURE 7:**
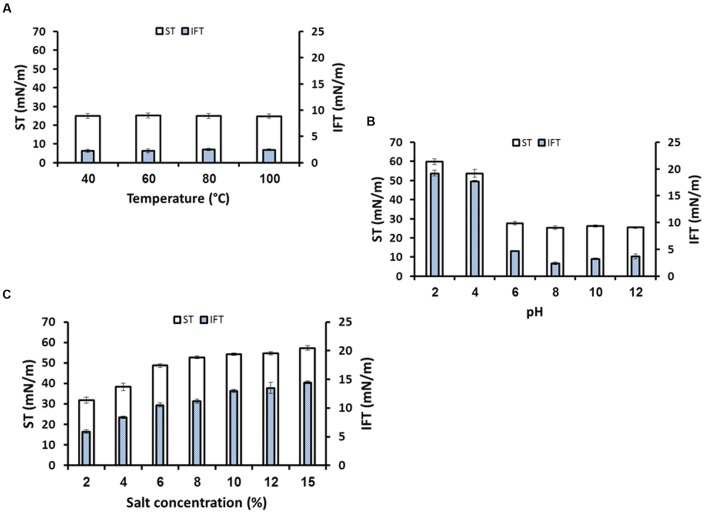
**The stability studies for biosurfactant produced by *B. licheniformis* W16, under different temperatures (A)**, pH **(B)**, and salinity **(C)**.

Any chemical agents to be used for EOR needs to be stable within a range of high temperatures (>40°C), wide range of pH and salinities. Therefore, the biosurfactant produced by *B. licheniformis* W16 was studied for its stability at wide range of harsh environmental conditions normally encountered in oil reservoirs. The biosurfactant was quite stable over a wide range of temperatures from 40 to 100°C (**Figure 7A**), and also was stable at temperatures higher than 100°C (121°C when subjected to autoclaving conditions; and at 100–160°C during drying-extraction using spray-dryer). The W16 biosurfactant was also stable in pH range of 6–12 and salt concentration up to 4% NaCl (**Figures 7B**, **C**). Under highly acidic pH (pH ≤ 4.0) biosurfactant was precipitated and thus higher ST and IFT values were observed. Several researchers also reported the stability of biosurfactants under high temperatures, at different pH values and salinities (Gudina et al., 2010; Khopade et al., 2012; Vaz et al., 2012; Saimmai et al., 2013; Al-Wahaibi et al., 2014; Jha et al., 2016).

Several mechanisms are reported for MEOR such as – selective plugging of highly porous rocks, reducing the viscosity of heavy oil by biodegradation, reduction of ST and IFT, wettability alteration etc. Previously, Al-Hattali et al. (2013) reported the potential of *B. licheniformis* W16 in enhancing the oil recovery in fractured carbonate rocks by growth under anaerobic conditions and selectively plugging the highly porous rock after 11 h incubation under reservoir conditions. They reported 27–30% additional oil recovery over residual oil saturation by microbial permeability profile modification (MPPM). In this study, the *ex situ* produced biosurfactant by *B. licheniformis* W16 was used for core-flooding studies using Berea sandstone core-plugs under reservoir conditions.

The salinity of the formation water was between 7 and 9%, pH ∼ 6.9, TDS ∼ 71000–72000 ppm, and major ions were (kg m^−3^): Sodium, 20.2–20.7; Calcium, 3.96–4.52; Magnesium, 0.95–1.02; Iron, 5; Chloride, 41.8–43.2; Sulfate, 0.33–0.35, Bromide, 0.30–0.31 (concentration of other ions and complete water chemistry can be found in Supplementary Table S1).

**FIGURE 8:**
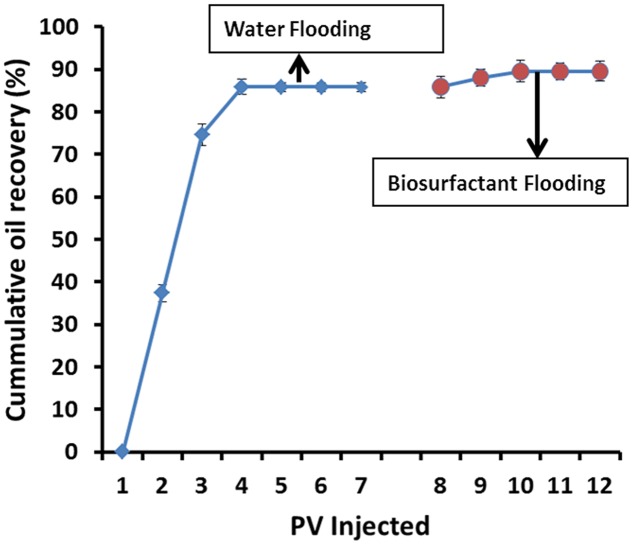
**The cumulative oil recovery (light oil – API 36.51°) from Berea sandstone core-plugs using biosurfactant**.

For core-flood experiments, cell-free biosurfactant broth was used. The pore volumes of the core-plugs were 17–19 cm^3^. Initial oil saturation (So_i_) in cores after oil flooding was around 73–77%, and after 7 PV of brine injection, nearly 80–85% (≅11.5 ml) of oil was produced and residual oil saturation (So_r_) was about 15–20%. Extra oil recovery was observed after injecting 4–5 PV of biosurfactant solution, where 24–26% (≅0.5 ml) of So_r_ was produced (**Figure 8**). Other researchers have reported 20–37% additional oil recovery over the residual oil saturation using cell-free biosurfactant injection in the core-plugs or sand-pack columns (Yakimov et al., 1997; Almeida et al., 2004; Al-Sulaimani et al., 2011a; Darvishi et al., 2011; Joshi and Desai, 2013; Al-Wahaibi et al., 2014; Arora et al., 2014; Elshafie et al., 2015; Joshi et al., 2015; Jha et al., 2016). In present study, the additional oil recovered could be due to mechanisms like reduction in ST/IFT and/or due to wettability alteration at the rock-oil-water interface, as observed during the course of study.

## Conclusion

To isolate *B. licheniformis* W16 produced a potent lipopeptide biosurfactant, within 48–72 h in carbohydrate based minimal media. It reduced ST, IFT and also altered the wettability thus changing the hydrophobic surface to more water-wet. To the best of our knowledge this is the first report of wettability alteration using biosurfactant produced by *B. licheniformis* strain. The biosurfactant produced by *B. licheniformis* W16 was characterized as lipopeptide, similar to lichenysin-A. It was quite stable under wide range of harsh reservoir conditions like high temperatures, pH range and salinities. It also successfully produced around 24–26% of residual oil (S_or_) from Berea sandstone core-plugs at 60°C. Further scale-up studies are recommended to better understand the economics of the biosurfactant applications at pilot-scale.

## Author Contributions

Conceived and designed the experiments: SJ, YA-W, SA-B, AE, ASA-B. Provided all the resources for performing experiments: YA-W, SA-B, AE, ASA-B, MA-M. Performed the experiments: SJ, AA-B. Analyzed the data: SJ, YA-W, SA-B, AE, ASA-B, AA-B. Drafting the work, revising it critically for important intellectual content, and final approval of the version to be published: all authors.

## Conflict of Interest Statement

The authors declare that the research was conducted in the absence of any commercial or financial relationships that could be construed as a potential conflict of interest.
