# Supplementary material for: Production, Characterization, and Application of Bacillus licheniformis W16 Biosurfactant in Enhancing Oil Recovery
Source: Front Microbiol. 2016 Nov 23;7:1853. doi: 10.3389/fmicb.2016.01853 (PMC5120096; doi:10.3389/fmicb.2016.01853)
Supplement: Supplementary file 1 [file Data_Sheet_1.pdf]

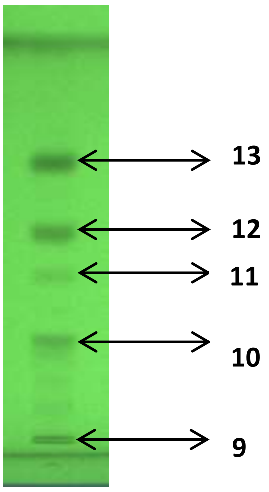


**Fig S1**: HPTLC plate developed with solvent system 4 (Butanol: Acetic acid: Water - 4:1:1). The bands 9 – 13 were eluted and further analyzed by ESI – MS, under positive and negative mode. The mass spectrum of each bands are as follows:

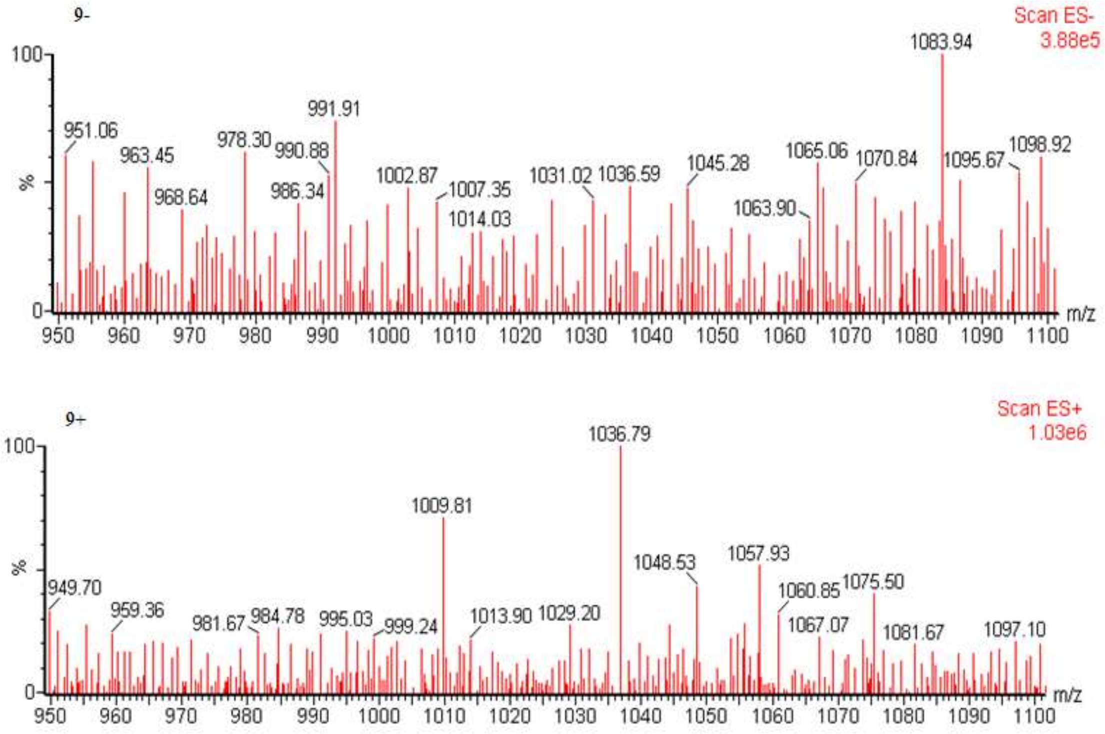


**ESI – MS of Band 9**

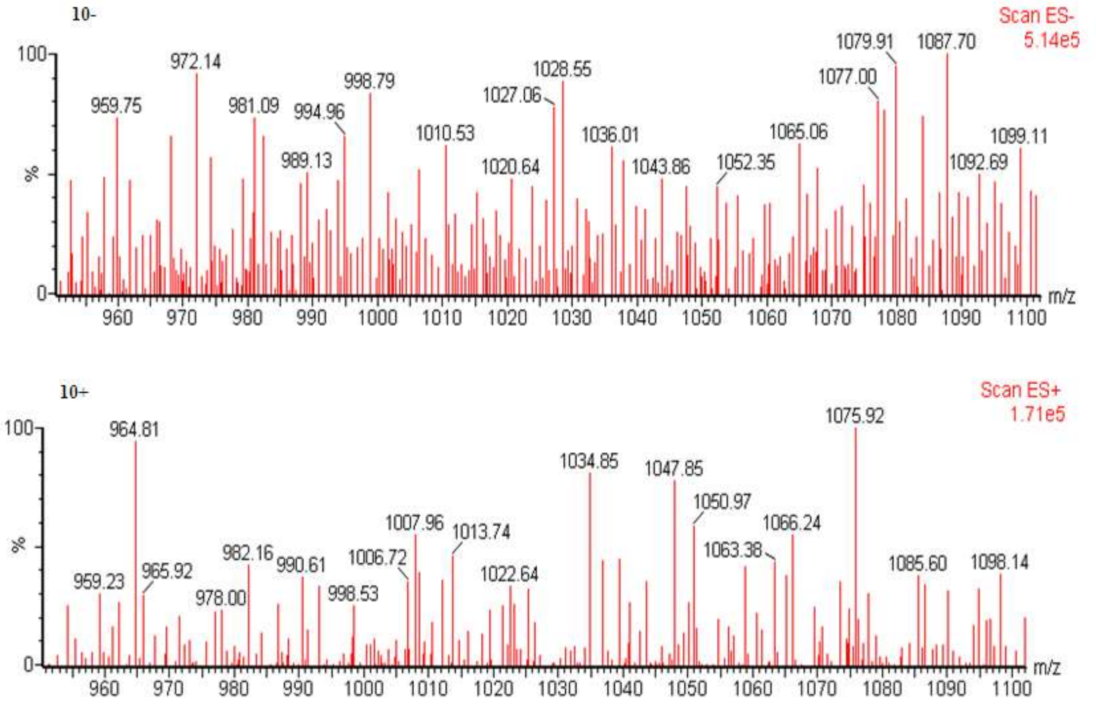


**ESI – MS of Band 10**

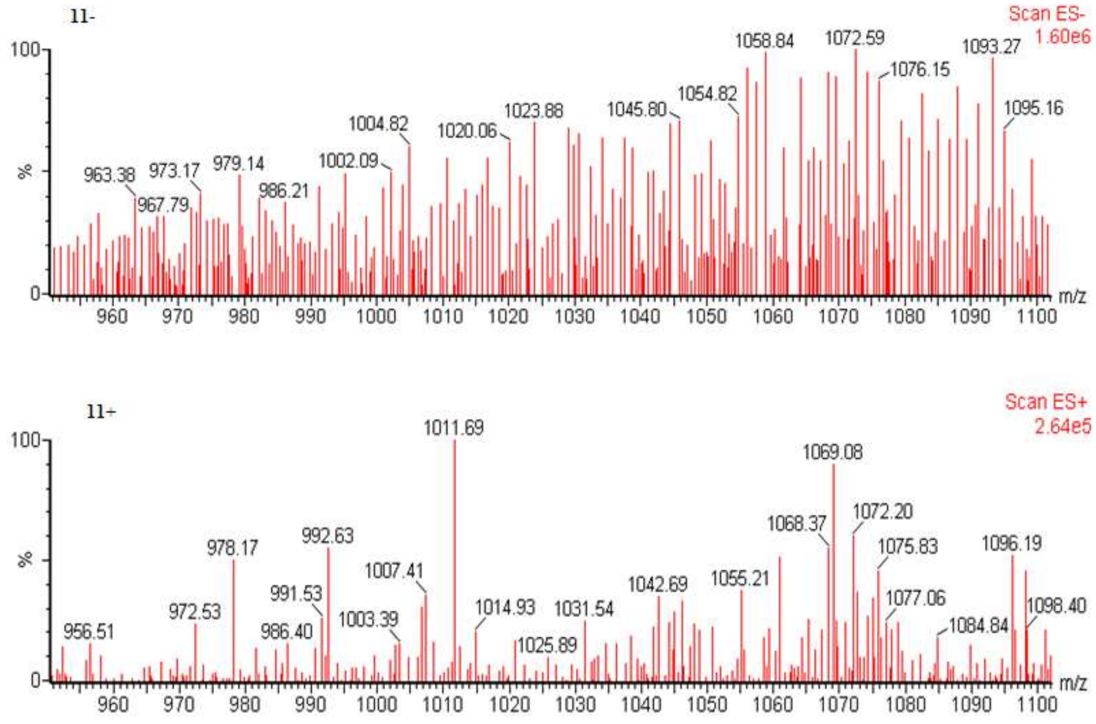


**ESI – MS of Band 11**

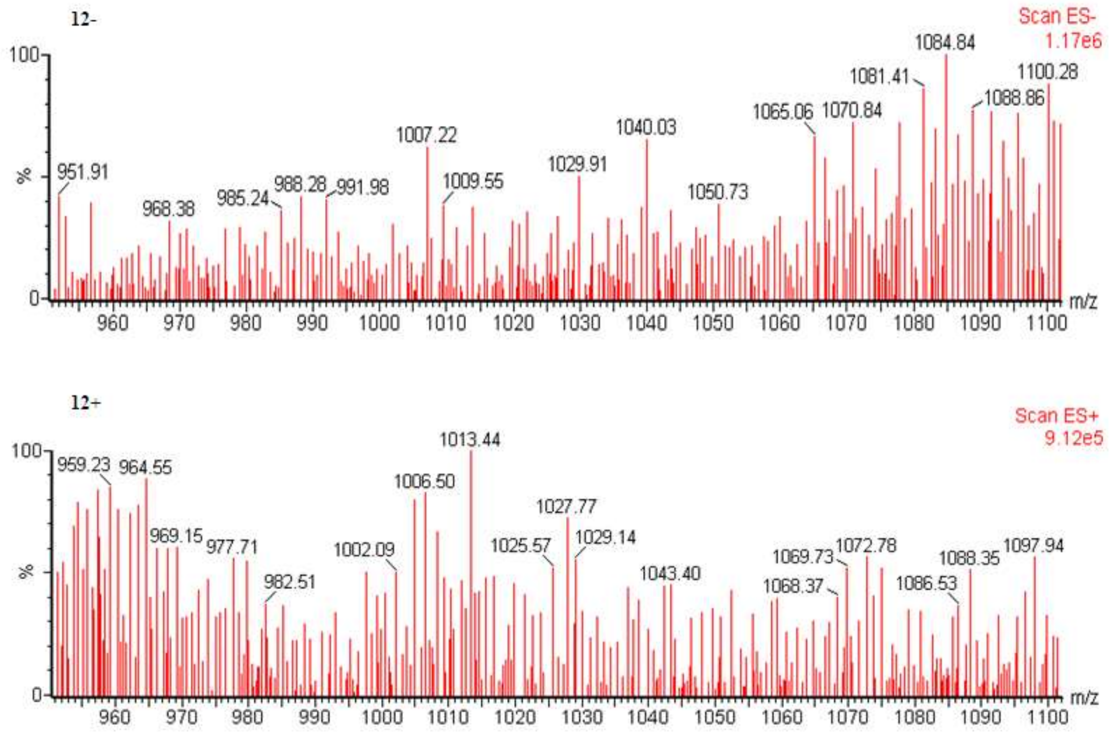


**ESI – MS of Band 12**

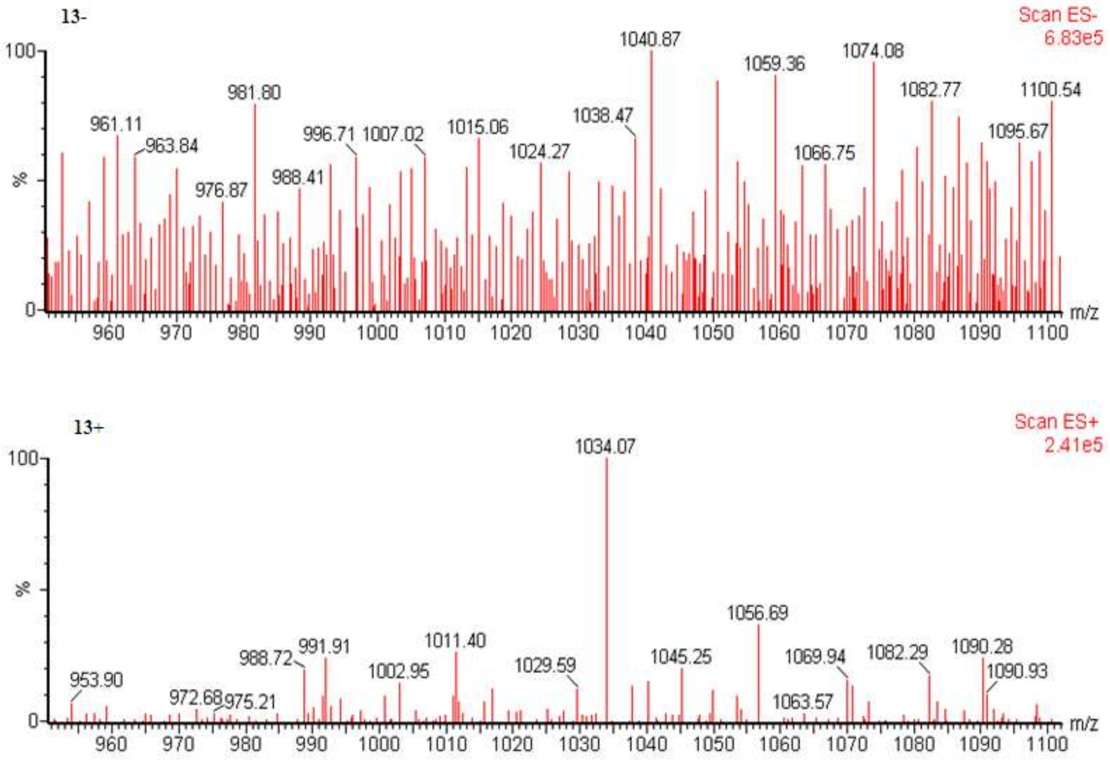


**ESI – MS of Band 13**

**Table S1: Formation water (FW) chemical analysis**

| Parameters | Samples | | |
|---|---|---|---|
| | **FW1#** | **FW2** | **FW3** |
| **Conductivity (µS/cm)** | **106200** | **107500** | **107700** |
| **Resistivity (Ω*cm)** | **9.40** | **9.30** | **9.28** |
| **Total Dissolved Solids (ppm)** | **71300** | **72000** | **72200** |
| **Temperature (°C)** | **20.9** | **20.9** | **20.9** |
| **pH** | **6.91** | **6.92** | **6.91** |
| **Specific gravity (@21 °C)** | **1.0645** | **1.0669** | **1.0631** |
| **Total Alkalinity (mg $CaCO_3$/L)** | **91** | **94** | **95** |
| **Turbidity (NTU)** | **48.4** | **50.9** | **55.5** |
| **Anions** | **FW1 (ppm)** | **FW2 (ppm)** | **FW3 (ppm)** |
| **F** | 1.44 | 1.13 | 0.99 |
| **Cl** | 42505.42 | 43248.84 | 41805.35 |
| **$NO_2$** | N.D. | N.D. | N.D. |
| **Br** | 308.73 | 315.92 | 302.78 |
| **$NO_3$** | 3.45 | 2.01 | 1.95 |
| **$PO_4$** | N.D.* | N.D. | N.D. |
| **$SO_4$** | 349.25 | 347.25 | 337.10 |
| **Cations** | **FW1 (ppm)** | **FW2 (ppm)** | **FW3 (ppm)** |
| **Li** | 8.83 | 4.86 | 5.71 |
| **Na** | 20566.49 | 20744.89 | 20191.39 |
| **$NH_3$** | N.D. | N.D. | N.D. |
| **K** | 172.86 | 166.97 | 161.46 |
| **Ca** | 3992.64 | 4271.56 | 4133.98 |
| **Mg** | 1023.84 | 986.71 | 953.66 |
| *These above 6 cations were analyzed by IC, and the ones below were analyzed by ICP-MS.* | | | |
| **Be** | <1 | <1 | <1 |
| **B** | <1 | <1 | <1 |
| **Al** | 11.2 | 12.2 | 6.5 |
| **V** | <5 | <5 | <5 |
| **Cr** | 10.2 | 11.5 | 13.5 |

| | | | |
|---|---|---|---|
| **Ni** | <5 | <5 | <5 |
| **Zn** | 7.4 | 11.5 | 12.4 |
| **Fe** | <5 | <5 | <5 |
| **As** | <1 | <1 | <1 |
| **Sr** | 68 | 132 | 75.8 |
| **Cd** | <5 | <5 | <5 |
| **Ba** | 176 | 165 | 157 |
| **Hg** | ND | ND | ND |
| **Pb** | <5 | <5 | <5 |

# FW1-FW3: Different formation water samples

*N.D.: Not detected
